# Network organization of resting-state cerebral hemodynamics and their aliasing contributions measured by functional near-infrared spectroscopy

**DOI:** 10.1088/1741-2552/acaccb

**Published:** 2023-01-18

**Authors:** Fan Zhang, Ali F Khan, Lei Ding, Han Yuan

**Affiliations:** 1 Stephenson School of Biomedical Engineering, The University of Oklahoma, Norman, OK 73019, United States of America; 2 Institute for Biomedical Engineering, Science and Technology, The University of Oklahoma, Norman, OK 73019, United States of America

**Keywords:** resting-state functional connectivity, functional near-infrared spectroscopy, diffuse optical tomography, default mode network, sampling rate, aliasing, aliased functional connectivity pattern

## Abstract

*Objective*. Spontaneous fluctuations of cerebral hemodynamics measured by functional magnetic resonance imaging (fMRI) are widely used to study the network organization of the brain. The temporal correlations among the ultra-slow, <0.1 Hz fluctuations across the brain regions are interpreted as functional connectivity maps and used for diagnostics of neurological disorders. However, despite the interest narrowed in the ultra-slow fluctuations, hemodynamic activity that exists beyond the ultra-slow frequency range could contribute to the functional connectivity, which remains unclear. *Approach*. In the present study, we have measured the brain-wide hemodynamics in the human participants with functional near-infrared spectroscopy (fNIRS) in a whole-head, cap-based and high-density montage at a sampling rate of 6.25 Hz. In addition, we have acquired resting state fMRI scans in the same group of participants for cross-modal evaluation of the connectivity maps. Then fNIRS data were deliberately down-sampled to a typical fMRI sampling rate of ∼0.5 Hz and the resulted differential connectivity maps were subject to a k-means clustering. *Main results*. Our diffuse optical topographical analysis of fNIRS data have revealed a default mode network (DMN) in the spontaneous deoxygenated and oxygenated hemoglobin changes, which remarkably resemble the same fMRI network derived from participants. Moreover, we have shown that the aliased activities in the down-sampled optical signals have altered the connectivity patterns, resulting in a network organization of aliased functional connectivity in the cerebral hemodynamics. *Significance.* The results have for the first time demonstrated that fNIRS as a broadly accessible modality can image the resting-state functional connectivity in the posterior midline, prefrontal and parietal structures of the DMN in the human brain, in a consistent pattern with fMRI. Further empowered by the fast sampling rate of fNIRS, our findings suggest the presence of aliased connectivity in the current understanding of the human brain organization.

## 1. Introduction

The brain remains remarkably active in the absence of explicit inputs (Shulman *et al* 2004). Since the early discovery that the hemodynamic oscillations in the functional magnetic resonance imaging (fMRI) data are temporally correlated at a resting state (Biswal *et al* 1995), a great deal of efforts have been dedicated to understanding these correlations as resting-state functional connectivity (RSFC) in studying human brain function and organization (Fox and Raichle 2007). For example, a widely-investigated default mode network (DMN) described as a network organization of correlated activities from medial prefrontal cortex (mPFC), posterior cingulate cortex (PCC)/precuneus, inferior parietal lobule, and lateral temporal cortex holds important roles for the normal neurocognitive functions of memory, consciousness and emotion regulation (Buckner *et al* 2008). Then the altered functional connectivity between anterior and posterior midline structures of DMN is found to be related to cognitive declining and has further been postulated as a hallmark of the aging process (Andrews-Hanna *et al* 2007) and Alzheimer’s Disease (AD) (Greicius *et al* 2004, Jones *et al* 2016), as well as implicated in other neuropsychiatric orders such depression and schizophrenia (Fox and Raichle 2007). The fMRI, alone or combined with electroencephalogram (EEG) or magnetoencephalography for better temporal resolution (Brookes *et al* 2011, Yuan *et al* 2012, Yuan *et al* 2016), has become the main tool for characterizing the functional organization of the human brain in healthy conditions, aging and developmental processes, and neurological disorders (Zhang and Raichle 2010).

Recently, an optics-based modality to noninvasively measure hemodynamics in the human brain—functional near-infrared spectroscopy (fNIRS)—has shown tremendous potential in studying RSFC (White *et al* 2009, Lu *et al* 2010, Zhang *et al* 2010, Chen *et al* 2020). fNIRS offers better cost-effectiveness, portability, and electromagnetic compatibility with medical devices (Scholkmann *et al* 2014, Naseer and Hong 2015, Chiarelli *et al* 2017, Hu *et al* 2020). Previous fNIRS studies have successfully mapped the resting state brain networks with seed-based correlation analysis or independent component analysis in different brain systems, such as prefrontal (Mesquita *et al* 2010), sensorimotor (White *et al* 2009, Lu *et al* 2010, Mesquita *et al* 2010), auditory (Lu *et al* 2010) and visual networks (White *et al* 2009, Mesquita *et al* 2010). Some recent studies have further validated the resting state brain networks obtained from fNIRS to those from fMRI via simultaneous fNIRS–fMRI recordings (Duan *et al* 2012, Sasai *et al* 2012). Noteworthy, these pioneer studies either utilized a sparse whole-head montage (Mesquita *et al* (2010) or used partial montages that only covered a part of the head (White *et al* 2009, Lu *et al* 2010, Duan *et al* 2012, Sasai *et al* 2012). Although the studies have indicated fNIRS’s capability of mapping functional connectivity in certain areas, whether fNIRS can image the higher-order large-scale brain networks, such as DMN that is important for neurological and neuropsychiatric disorders (Andrews-Hanna *et al* 2007, Greicius 2008, Zhang and Raichle 2010), is yet to be seen. Especially the imaging of DMN is emphasized, since it provides critical information on the normal aging trajectory and the pathological deviation towards diseased stages of AD (Ittner and Götz 2011, Karran *et al* 2011, Beard *et al* 2016, Edwards 2019), and further holds pivotal positions of biomarkers in clinical trials of intervention and prevention (Sperling *et al* 2014, Cummings *et al* 2016, McDade *et al* 2021). Considering the overwhelming number of people that are projected to be with AD and an even larger number to be placed at the prevention and intervention stages, a broadly accessible instrument such as fNIRS that can image and monitor the network organization of DMN and other resting-state networks (RSN) is especially welcomed (Chen *et al* 2022).

However, traditional fNIRS only provides topographic information on brain activity and has a limited spatial resolution (Obrig and Villringer 2003). The tomographic reconstruction of fNIRS, namely diffuse optical tomography (DOT), can integrate MRI structural images and provide a much refined millimeter-level spatial resolution, therefore attracting great interest in research and clinics (Culver *et al* 2003, Boas *et al* 2004). Extended from an early DOT study mapping the sensorimotor and visual networks (White *et al* 2009), a recent study utilizing a high-density configuration of optodes has successfully mapped the large-scale cognitive networks of dorsal attention, fronto-parietal and partially DMN in a group of healthy subjects (Eggebrecht *et al* 2014). However, two important regions in DMN—mPFC and posterior midline structures—are missing due to the absence of the optode array coverage. Another DOT study with two high-density patches of optodes covering the frontal and posterior areas has reported DOT RSFC as part of DMN, which however was still incomplete and missed the bilateral inferior parietal lobes (IPL) (Aihara *et al* 2020). Recent efforts from our own group have established whole-head and cap-based fNIRS recordings and have shown our capability of DOT to reconstruct brain-wide, voxel-wise activity (Chen *et al* 2020, Khan *et al* 2021, Zhang *et al* 2021), yet RSFC of a complete DMN by DOT has not been fully revealed and systematically benchmarked to fMRI DMN.

In addition, fNIRS offers a high temporal resolution, typically at the rate of 2–10 Hz (Eggebrecht *et al* 2014, Chen *et al* 2020, Zhang *et al* 2021), which is higher than a typical fMRI whole-brain scan of 0.5 Hz (Andrews-Hanna *et al* 2007, Yeo *et al* 2011). Despite intrinsic oscillations of the vascular networks (Mateo *et al* 2017, Drew *et al* 2020) and other physiological oscillations such as respiration, cardiac pulsation and cerebrospinal fluid that may also contribute to the measured hemodynamics in the upper frequencies (Liu 2016), our understanding of the human brain functional connectivity has been primarily established by fMRI at sub-hertz sampling rates for the activities below 0.1 Hz (Biswal *et al* 1996, Mitra *et al* 1997, Cordes *et al* 2001). Since hemodynamics are resulted from the interplay between local neuronal activity, oxygen consumption, and vascular circulation (Logothetis 2008, Buxton 2013), it is critical to understand how the oscillations that exist beyond those safely sampled range could contribute to the network organization via the aliased activities. Recent studies have utilized techniques such as magnetic resonance encephalography (Huotari *et al* 2019), multiband sequence (Tong and Frederick 2014, Golestani *et al* 2017, Demetriou *et al* 2018), or slice-accelerated protocol (Cordes *et al* 2014, Golestani *et al* 2017) to study the aliasing effect due to insufficient sampling rates and the spatial distribution of physiological, non-neuronal contributions. Yet, the effect of sampling rate on imaging the functional connectivity of DMN remains elusive—some study reported consistent patterns at a lowered sampling rate (Huotari *et al* 2019), while others have noted strong contributions from respiration or cardiac pulsation (Cordes *et al* 2014). In this context, DOT, which has a higher sampling rate compared with fMRI and can critically sample the cerebral hemodynamic signals to avoid the aliasing of cardiorespiratory activity into low-frequency oscillations, can be used to study the sampling effects on RSFC.

In the present study, we aimed to validate the whole-head fNIRS capability in mapping distributed functional connectivity via comparison with fMRI, and also to characterize the aliasing effects on functional connectivity due to an insufficient sampling rate. To achieve these goals, we used a whole-head, cap-based and high-density fNIRS array to measure oxyhemoglobin (HbO) and deoxyhemoglobin (HbR) at the sampling frequency of 6.25 Hz. By computing DOT based on individuals’ realistic anatomy, we have mapped the network at a fine voxel resolution to a standard cortical surface space, and then benchmarked fNIRS maps of DMN to those obtained from fMRI. Furthermore, we deliberately down-sampled the raw optical recordings to a sub-hertz temporal resolution and have examined the altered connectivity in the ultra-slow frequency range with regard to the seeds located within DMN.

## 2. Method

### 2.1. Participants

The study was approved by the University of Oklahoma Health Sciences Center Institutional Review Board. Written consent was obtained from all participants prior to the study. All procedures were carried out in accordance with the IRB guidelines. A total of 20 healthy participants without any neurological or neuropsychiatric disorders were screened and enrolled in this study. Three participants were not able to complete all data recordings. In addition, resting-state data from four participants were excluded from the present study due to poor data quality (primarily excessive motions). Therefore, data from thirteen participants (five females, 31.7 ± 9.3 years old) were used for the analyses. Participants received financial compensation for their participation.

### 2.2. Data acquisition

fMRI data of the blood-oxygenation-level-dependent (BOLD) contrast and fNIRS–EEG data were acquired from each participant in two separate visits. Structural head MRI and fMRI images of participants were acquired using a GE Discovery MR750 whole-body 3-Tesla MRI scanner (GE Health, Milwaukee, WI, USA). The following parameters were used for MRI scanning: field of view (FOV) = 240 mm, axial slices per slab = 180, slice thickness = 1 mm, image matrix = 256 × 256, Repetition Time / Echo Time (TR/TE) = 8.45/3.24 ms. The following parameters were used for fMRI scanning: FOV = 240 mm, axial slices per slab = 41, slice thickness = 4 mm, image matrix = 64 × 64, TR/TE = 2600/60 ms. fMRI data were recorded when participants rested in a supine position inside the scanner with their eyes opened. One 6-minute resting session per participant was included for the fMRI.

**Figure 1.**
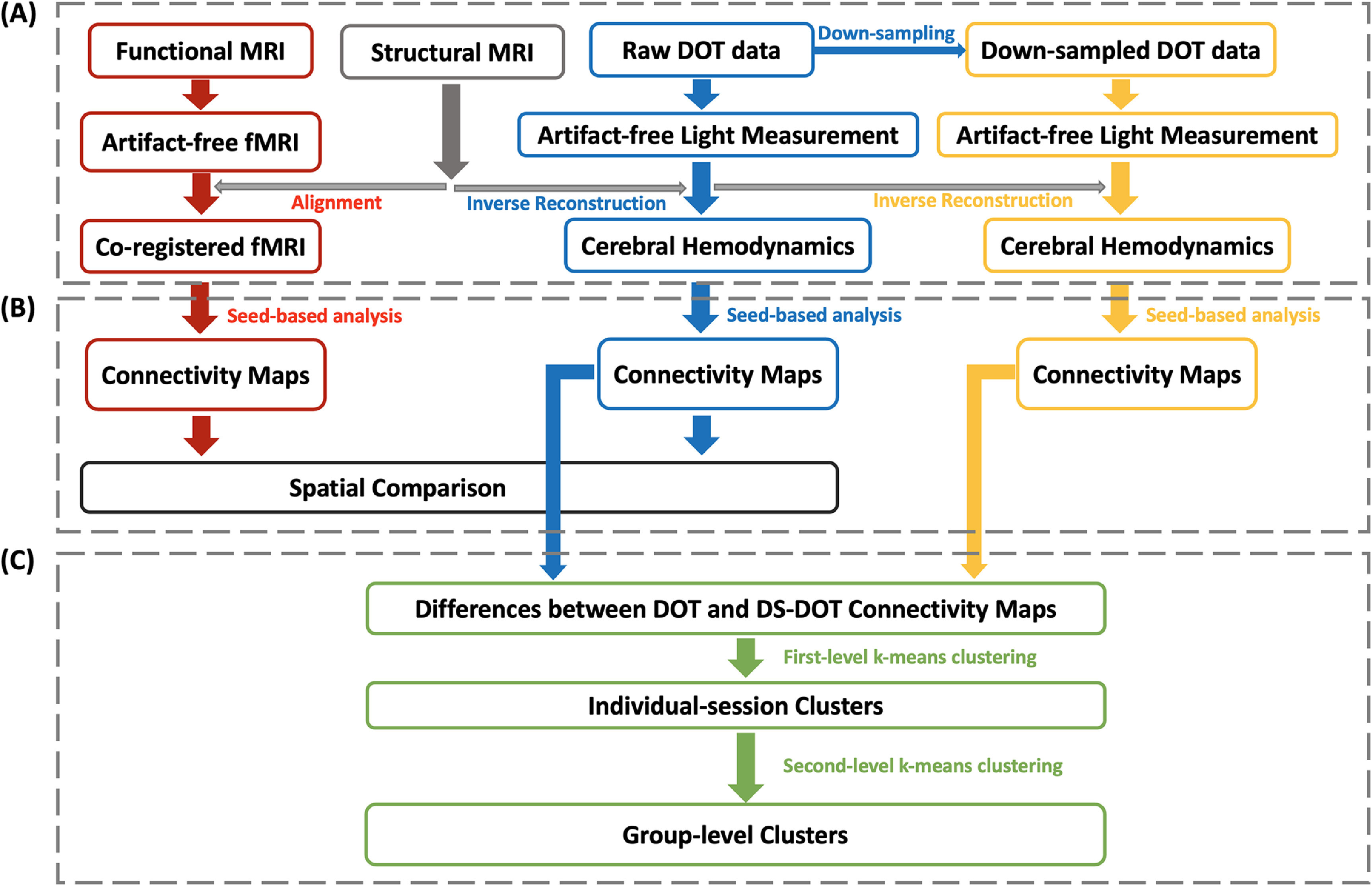
Schematic framework of computations. (A) Data preprocessing of fMRI, fNIRS and down-sampled fNIRS data. (B) Seed-based functional connectivity analyses. (C) *K*-means clustering on the differential functional connectivity maps between fNIRS and down-sampled fNIRS.

Simultaneous fNIRS, EEG (not analyzed in this study) and peripheral measurements of respiration, pulse, and triaxial acceleration were recorded, following the described protocol (Zhang *et al* 2021). The whole-head montage of optodes for the data analyzed in current study was described the figure 1 of our previous publication (Zhang *et al* 2021). Two 6-minute recording sessions of eyes-open resting state were acquired per participant who comfortably sit in a dark and sound-damped room.

### 2.3. fNIRS data preprocessing

fNIRS data were automatically preprocessed by adapting an automatic denoising procedure, namely principal-component-analysis-based general linear model (PCA-GLM) (Zhang *et al* 2021). As illustrated in figure 1, the preprocessing pipeline included these steps: (a) converting raw data to optical density (OD); (b) computing power spectral densities using the Welch’s method (a window length of 60 s and 50% overlap) and excluding bad channels that showed no heartbeat frequency peak (0.8–1.6 Hz); (c) bandpass filtering OD with 0.008–0.2 Hz; (d) identifying and rejecting bad time segments with excessive head movements using the metric of global variance in temporal derivative (GVTD) (Sherafati *et al* 2020). The time points that had GVTD values larger than three times the mean GVTD value across all time points were identified, and then time windows of 10 s centered at these time points were masked as bad time segments and excluded from further analyses (Sherafati *et al* 2020). (e) Although fNIRS has its intrinsic advantage of high sampling frequency, interferences from auto-regulation in the vasculature and other physiological sources occur at various frequencies (White *et al* 2009). By employing eight short-separation (SS) channels that have minimal penetration into the cortical tissue, we measured the superficial hemodynamics in the scalp and used that combined with long-separation (LS) channels to construct a superficial signal regressor, which was then removed as one of the nuisances. Specifically, PCA was applied to LS OD data to decompose signals into multiple components. The spatial uniformity of each principal component was assessed by the metric of coefficient of spatial uniformity (Kohno *et al* 2007). Because spatial uniformity indicates superficial skin responses (Zhang *et al* 2005), a principal component with the highest value of spatial uniformity was identified, namely PC-LS. Then, instead of directly removing the time course of PC-LS from the multi-channel data, the PC-LS was used to select a component from the SS measurements which capture the absorption by superficial tissues (Saager and Berger 2005). A total of eight, evenly distributed SS OD data were subject to a separate PCA, and the SS component that had the highest temporal correlation with PC-LS was chosen, representing the skin response. The selected SS component was then used as a nuisance regressor to be removed by GLM. (f) A GLM was configurated per session to remove physiological noise. Prior to GLM, auxiliary data of acceleration, respiration, cardiac pulsation were processed by a band-pass filter with 0.008 Hz–0.2 Hz, detrended by a 3-order polynomial drift and re-sampled to 6.25 Hz (originally acquired at 500 Hz) by a decimation ratio of 80. The time course of the SS component, the detrended auxiliary data (acceleration, respiration and cardiac pulsation) and a 3-order polynomial drift were included in the design matrix as nuisance regressors.

In addition, in order to investigate the effect of a low sampling rate on functional connectivity, we have re-sampled the fNIRS data (originally acquired at 6.25 Hz) by a decimation ratio of 13, resulting in a sampling rate of 0.48 Hz. The down-sampled fNIRS data were preprocessed using the same procedure. For applying PCA–GLM on the down-sampled fNIRS data, the auxiliary data of acceleration, respiration, and cardiac pulsation were similarly processed by a band-pass filter with 0.008 Hz–0.2 Hz, detrended by a 3-order polynomial drift, and then re-sampled to 0.48 Hz, i.e. the same rate as the down-sampled fNIRS.

### 2.4. DOT computation

Participant-specific structural MRI data were used to create individual finite element method (FEM) volume meshes (Khan *et al* 2021). A spectrally-constrained linear FEM-based forward model (Srinivasan *et al* 2005) linking hemodynamic responses $\overset{{\scriptscriptstyle\rightharpoonup}} {x} \left( t \right)$ and scalp-based light measurements $\overset{{\scriptscriptstyle\rightharpoonup}} {y} \left( t \right)$ was established as $\overset{{\scriptscriptstyle\rightharpoonup}} {y} \left( t \right) = A\overset{{\scriptscriptstyle\rightharpoonup}} {x} \left( t \right)$, where the sensitivity matrix $A$ was calculated using the NIRFAST software (Dehghani *et al* 2009a). The volumetric inverse source space was constructed with nodes inside the brain (enclosed by the pial surface) and no deeper than 45 mm from the scalp, beyond which the sensitivity had dramatically decreased. A single-step joint inverse reconstruction using regularized minimum norm estimate (Bertero *et al* 1988) yielded the volumetric inverse solution data $\overset{{\scriptscriptstyle\rightharpoonup}} {x} \left( t \right)$. The inverse data were first smoothed with a 6 mm spherical kernel and then projected to the fsaverage5 smoothed white matter surface from FREESURFER (Ségonne *et al* 2004, Fischl 2012). The projection was performed by assigning the data to each node on the cortical surface from the nearest finite element node in terms of Euclidean distance. Both cortical data of HbO and HbR concentration changes were reconstructed. Because the deoxygenated status reflected in HbR is a direct approximation to the T2* contrast in fMRI (Ogawa *et al* 1990), HbR was primarily used in the further analysis of this study for the benchmark purpose. Tomography for the down-sampled fNIRS data was computed in the same procedure, resulting in down-sampled DOT (DS-DOT). The DOT and DS-DOT data were subject to band-pass filtering of 0.009 Hz–0.08 Hz which is commonly used in fMRI RSFC analysis.

### 2.5. fMRI preprocessing

For the purpose of cross-modal validation, we have also collected resting-state fMRI data in the same group of participants. The fMRI data processing was performed using Analysis of Functional NeuroImages software (AFNI, http://afni.nimh.nig.gov/) (Cox 1996). The first five volumes of each fMRI run were excluded from the analysis to allow the BOLD signal to reach a steady state. The fMRI then went through these processing steps including slice timing and rigid-body motion correction, spatial smoothing with a Gaussian kernel with a full width at half maximum (FWHM) of 13 mm, and a bandpass filtering (0.009–0.08 Hz). Notably, the 13 mm FWHM was considered to match up with that in DOT. In addition, six affine motion parameters and the global signal from the entire brain were regressed from the dataset. Data points of excessive motion (root mean square larger than 0.2 mm) were excluded from regression and further analysis using the *censoring* option implemented in AFNI (afni_proc.py). The threshold of 0.2 mm was suggested by Power and colleagues (Power *et al* 2013). Specifically, the L2-norm of the derivatives of the motion parameters estimated from motion registration was calculated and those time points with an L2-norm larger than the threshold of 0.2 mm were censored/excluded in the regression and the later functional connectivity analysis. The fMRI data of each participant were firstly spatially co-registered to high-resolution individual anatomical images and then normalized to the Talairach and Tournoux template brain (Talairach and Tournoux 1988).

### 2.6. Seed-based mapping of resting-state functional connectivity

After preprocessing, seed-based mapping of functional connectivity was performed in fMRI, DOT and DS-DOT by calculating the Pearson’s correlation coefficients between the average time course of a seed region with a radius of 10 mm and the time courses of other nodes (Power *et al* 2011). In order to study the functional connectivity maps of DMN, we considered the key nodes of DMN as the seed regions, including mPFC, IPL, precuneus and a compound seed (the average of mPFC, IPL, and precuneus). Table 1 lists the coordinates of the seeds.

**Table 1.** The coordinates of seeds for the resting state functional connectivity maps. All numbers are in Montreal Neurological Institute (MNI) coordinates. Identical seeds are used for fNIRS and fMRI maps.

	Left hemisphere			Right hemisphere		
	*x*	*y*	*z*	*x*	*y*	*z*
mPFC	−8.4	51.5	14.9	8.7	52.4	15.1
IPL	−39.5	−57.0	40.7	39.0	−55.9	42.6
Precuneus	−6.5	−64.5	39.8	6.6	−62.5	39.6

mPFC: medial prefrontal cortex. IPL: inferior parietal lobule.

For fMRI data, the correlation values were first subject to Fisher’s Z transform. For the cross-modal evaluation, we projected the functional connectivity to a common space of the cortical surface. Specifically, the individual-level volumetric Z maps were projected to the fsaverage5 smoothed white matter surface with 10 242 vertices per hemisphere (Fischl *et al* 1999) using the *3dVol2Surf* in AFNI. The individual-level *Z* maps in the surface space were then averaged to obtain the group-level connectivity map and subject to node-wise one-sample two-tailed Student’s t-tests to assess the significance of functional connectivity.

For DOT and DS-DOT data, the global signal was first calculated by averaging the time traces of all nodes over the cortical surface and regressed out from the time traces of individual cortical nodes (Desjardins *et al* 2001). The seed-based correlation analysis was then performed in the surface space. The individual maps of Pearson’s correlation coefficients were subject to Fisher’s Z transform and smoothed with a Gaussian kernel with FWHM of 13 mm using the mri_surf2surf in FreeSurfer (Dale *et al* 1999). The smoothed individual-level *Z* surface maps were then averaged to obtain the group-level connectivity map.

The similarity between the DOT and fMRI functional connectivity was quantitatively evaluated by spatial correlation and dice coefficient (Eggebrecht *et al* 2014, Yuan *et al* 2016). The spatial correlation was calculated for the unthresholded DOT and fMRI functional connectivity maps with the same seed. The dice coefficient was to evaluate the similarity between binary images based on the binary DOT and fMRI maps after thresholding (1 for a significant *p*-value after multiple-comparison correction and 0 for non-significant). The dice coefficient was calculated as: Dice = 2|A∩B|/(|A|+|B|), where A represents the binary DOT map, B represents the binary fMRI map, and | ⋅ | represents the cardinal of set A or B.

### 2.7. Aliased functional connectivity patterns (AFCP)

In order to study the effect of aliased sampling on the RSFC, we have down-sampled the fNIRS data at the rate of 0.48 Hz. After identical procedures of preprocessing and tomography projection, functional connectivity maps were created based on DOT and DS-DOT, respectively. Differential connectivity maps were calculated by subtracting the DOT connectivity maps from the DS-DOT connectivity maps. Since aliased activity led to aliased connectivity maps, we explored whether any structural organization exists in these differential connectivity maps. Because the secondary goal of this study was to examine whether the DMN was in any way affected by an insufficient sampling rate, we used a data-driven approach to search for organized patterns among all the differential maps associated with nodes in the DMN. A two-level *k*-means clustering was performed on the differential functional connectivity maps based on their spatial similarities (Britz *et al* 2010, Michel and Koenig 2018). At each level, the clustering was repeated 100 times to increase the chances of escaping local minima, with randomly initialized centroid positions (Allen *et al* 2014). In terms of the distance measure, we selected the ‘cosine’ distance to consider the sign of these differential values with an emphasis on the overall similarity among patterns.

Specifically, each node in the Yeo template of DMN (Yeo *et al* 2011) was selected as a seed, resulting in a differential connectivity map associated with the seed. The individual-session differential maps were subject to the first-level *k*-means clustering. Similar to previous studies, the number of clusters was determined using the criterion of the cluster cost, computed as the ratio between within-cluster distance to between-cluster distance (Allen *et al* 2014, Li *et al* 2021), in order to minimize the within-cluster distance and maximize the between-cluster distance. The appropriate number of k was selected at the elbow of the curve, which optimally balances the cluster cost and cluster number. The individual-session centroids were then concatenated and used as input for the second-level clustering to yield group-level patterns which are in the following referred to as AFCP.

Furthermore, we utilized a region-of-interest (ROI) approach to visualize which frequencies contributed to the aliased connectivity values. Based on each AFCP, we have selected a seed ROI that yielded the corresponding AFCP map and then selected a target ROI in the hemisphere-symmetric location with representative differential values. We quantified the frequency contribution to aliased connectivity by decomposing the correlation coefficient between time traces of the seed and target ROIs by Fourier transformation of the time traces (Cordes *et al* 2000, 2001). Three frequency bins were calculated: <0.05 Hz, 0.05–0.1 Hz, 0.1–0.2 Hz, for both DOT and DS-DOT. Note that AFCP were calculated based on the DOT and DS-DOT filtered from 0.009 Hz to 0.08 Hz. Therefore, we have normalized the frequency-specific connectivity values at each bin to the connectivity values of 0.009–0.08 Hz in original DOT, in order to itemize the frequency-specific contributions.

### 2.8. Statistical analysis

To assess the significance of the functional connectivity values, all group-level maps were subject to node-wise one-sample two-tailed Student’s t-tests. The resulted *p* values were corrected for multiple comparisons using Benjamini–Hochberg method (*n* = 12 021; *n* is the number of nodes which are commonly available across all participants) (Benjamini and Hochberg 1995), resulting in *q* values. Also, to assess the significance of the *AFCP*, all group-level centroids were subject to node-wise one-sample two-tailed Student’s t-tests. The resulted statistics were also corrected for multiple comparisons using Benjamini–Hochberg method (Benjamini and Hochberg 1995).

## 3. Results

### 3.1. Hemodynamic activity at sensor level

**Figure 2.**
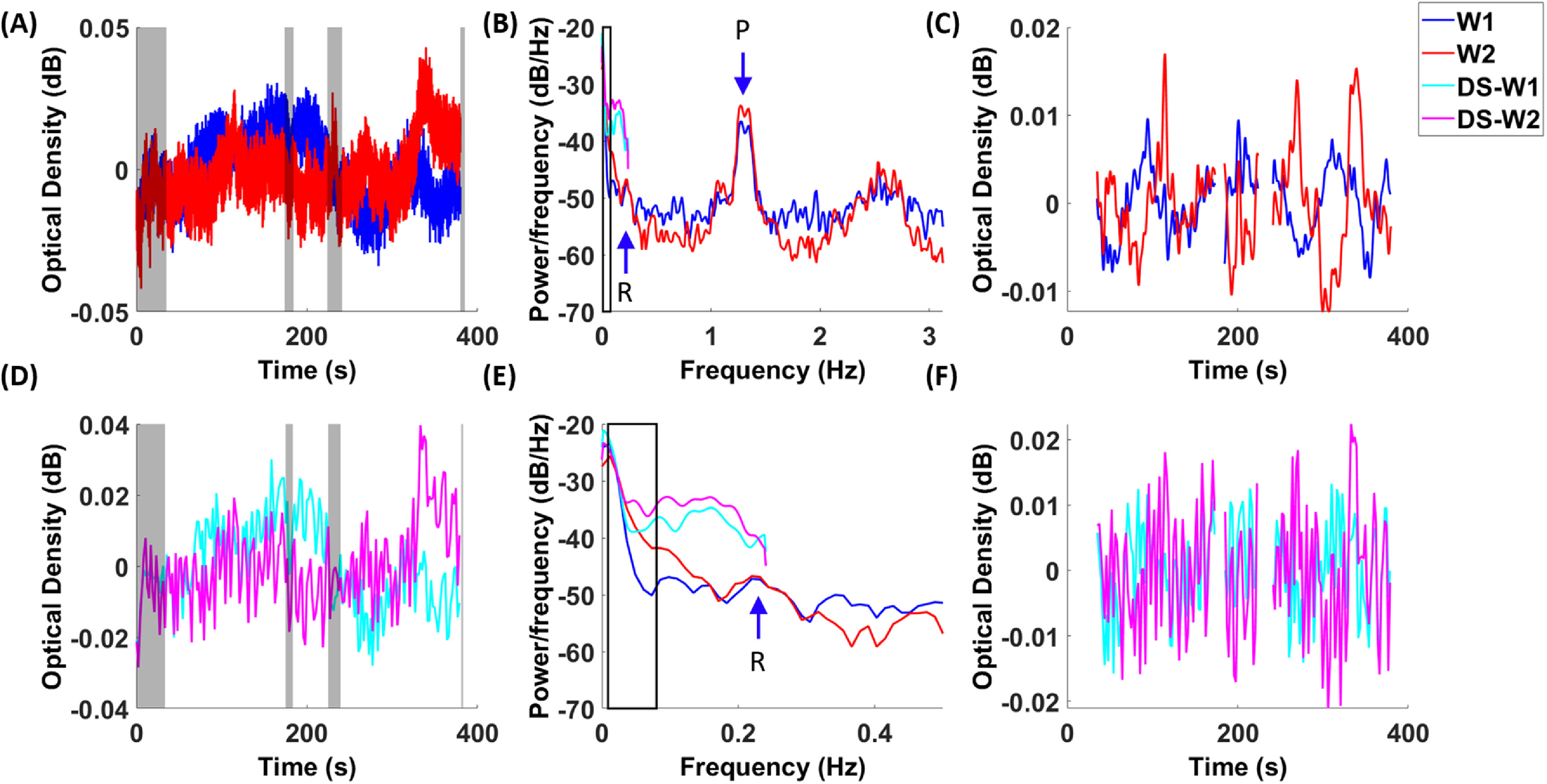
fNIRS time courses and spectrums. Temporal traces of raw data in fNIRS (A) and down-sampled fNIRS (D) are plotted in two wavelengths (W1: 760 nm, W2: 850 nm), with gray shaded areas indicating the rejected time segments with motion artifacts. Power spectrums of fNIRS and down-sampled fNIRS are shown in (B) and (E), with black squares indicating the range from 0.009 to 0.08 Hz. Panel (E) is the enlarged portion of Panel (B) from 0 to 0.5 Hz. Band-pass filtered time courses in fNIRS (C) and down-sampled fNIRS (F) are illustrated. An arrow labeled with ‘P’ indicates the peak associated with cardiac pulses in the signal. An arrow labeled with ‘R’ indicates the peak associated with respirations in the signal. Red and blue lines indicate originally sampled data, while pink and cyan lines indicate down-sampled data.

In the present study, resting-state hemodynamic activity in the human participants are measured by pairing optical sources and detectors in a whole-head, cap-based system. The time traces of a fNIRS channel in two wavelengths (760 nm and 850 nm) are illustrated in figure 2(A), with time segments of motion artifacts that are excluded from further analyses. The power spectrums of the time traces are shown in figure 2(B). Notably in the representative participant, the cerebral hemodynamics show a peak in the range of < 0.1 Hz, in addition to peaks associated with the cardiac pulse (∼1.3 Hz) and respiration (∼0.23 Hz). After band-pass filtering of 0.008–0.2 Hz, the time traces present resting-state, spontaneous fluctuations in figure 2(C).

In addition, in order to examine the effect of a low sampling rate on the hemodynamic activity and connectivity, we have deliberately down-sampled the time traces of the fNIRS data from originally 6.25 Hz to 0.48 Hz (figure 2(D)). As a result, there are considerable differences in the amplitudes of PSDs between fNIRS and down-sampled data (figure 2(E)). Notably, down-sampled data present higher power amplitudes than the original data, due to the aliasing of cardiac and respiratory activities into the ultra-slow range. After band-pass filtering of 0.008–0.2 Hz, there appears a greater amount of and faster-evolving fluctuations in the down-sampled data (figure 2(F)).

### 3.2. RSFC maps between DOT and fMRI

**Figure 3.**
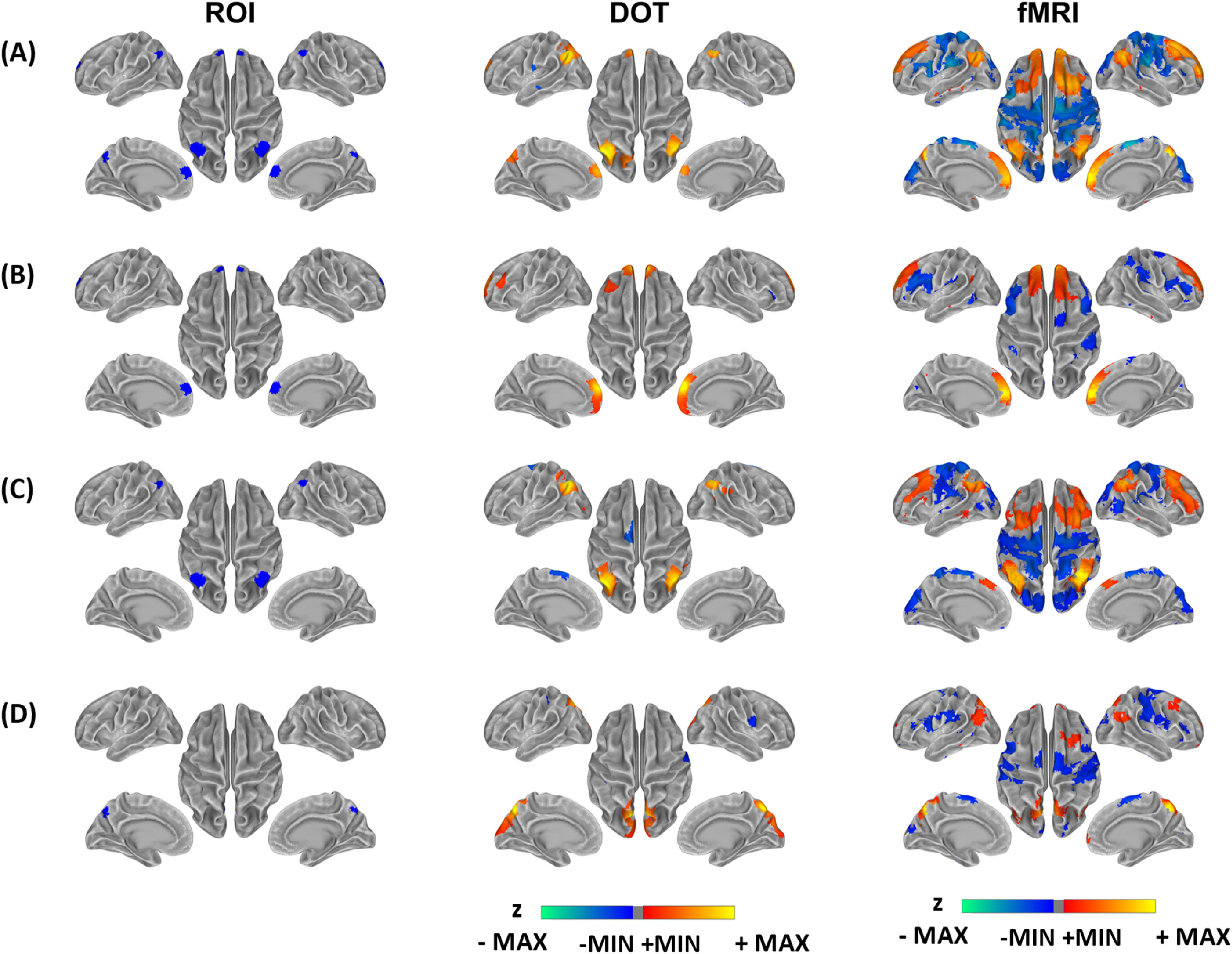
Resting state functional connectivity by fNIRS HbR and fMRI. Connectivity maps are based on seeds of (A) a compound seed (the average of mPFC, IPL and precuneus) (B) mPFC, (C) IPL and (D) precuneus. All maps are thresholded according to *q* < 0.05 after multiple comparison correction. Walm colors from red to yellow indicate the positive *z* values after thresholding. Cold colors from blue to green indicate the negative *z* values after thresholding. The threshdolded connectivity maps are overlaid on the standard cortical surface with grey colors indicating the curvature profile of the surface. mPFC: medial prefrontal cortex, IPL: inferor parietal lobules.

We evaluated the cross-modal correspondence of DOT and fMRI regarding the default mode network obtained in the same group of participants. The seed regions and corresponding seed-based functional connectivity maps with mPFC, IPL, precuneus and a compound seed (the average of mPFC, IPL, and precuneus) are shown in figure 3, with maps thresholded at *q* < 0.05 after multiple-comparison correction. Supplemental figure 1 shows the unthresholded maps. The seed-based functional connectivity maps of DOT in both figure 3 and supplemental figure 1 present spatial patterns comparable with those of fMRI, which is consistent with high spatial correlations and dice coefficient values between DOT and fMRI functional connectivity maps, as listed in table 2. The compound-seeded functional connectivity maps of DOT depict the DMN hubs including mPFC, IPL, and precuneus, which spatially resembles to those of fMRI (figure 3(A)). The mPFC-seeded functional connectivity maps of DOT show functional connectivity between left and right mPFC, similar to that of fMRI (figure 3(B)). The IPL-seeded functional connectivity maps of DOT exhibit similar spatial patterns to that of fMRI and reveal functional connectivity between left and right IPL (figure 3(C)). The precuneus-seeded functional connectivity maps of DOT show functional connectivity at left and right precuneus as depicted in fMRI (figure 3(D)). Since the HbR contrast approximates the imaging mechanism of fMRI, we primarily focused our cross-modal investigation on HbR data. In addition, supplemental figure 2 shows the HbO-derived default mode network that also presents a consistently high spatial resemblance with fMRI, which is consistent with the quantitative values of spatial correlations and dice coefficients listed in supplemental table 1.

**Table 2.** Comparison between resting state functional connectivity maps by fNIRS HbR and fMRI. Spatial correlations are calculated based on unthresholded maps. Dice coefficients are calculated based on thresholded maps at *q* < 0.05 after multiple-comparison correction.

	Spatial correlations	Dice coefficients
Compound	0.47	0.20
mPFC	0.56	0.29
IPL	0.49	0.22
Precuneus	0.57	0.23

### 3.3. AFCP

**Figure 4.**
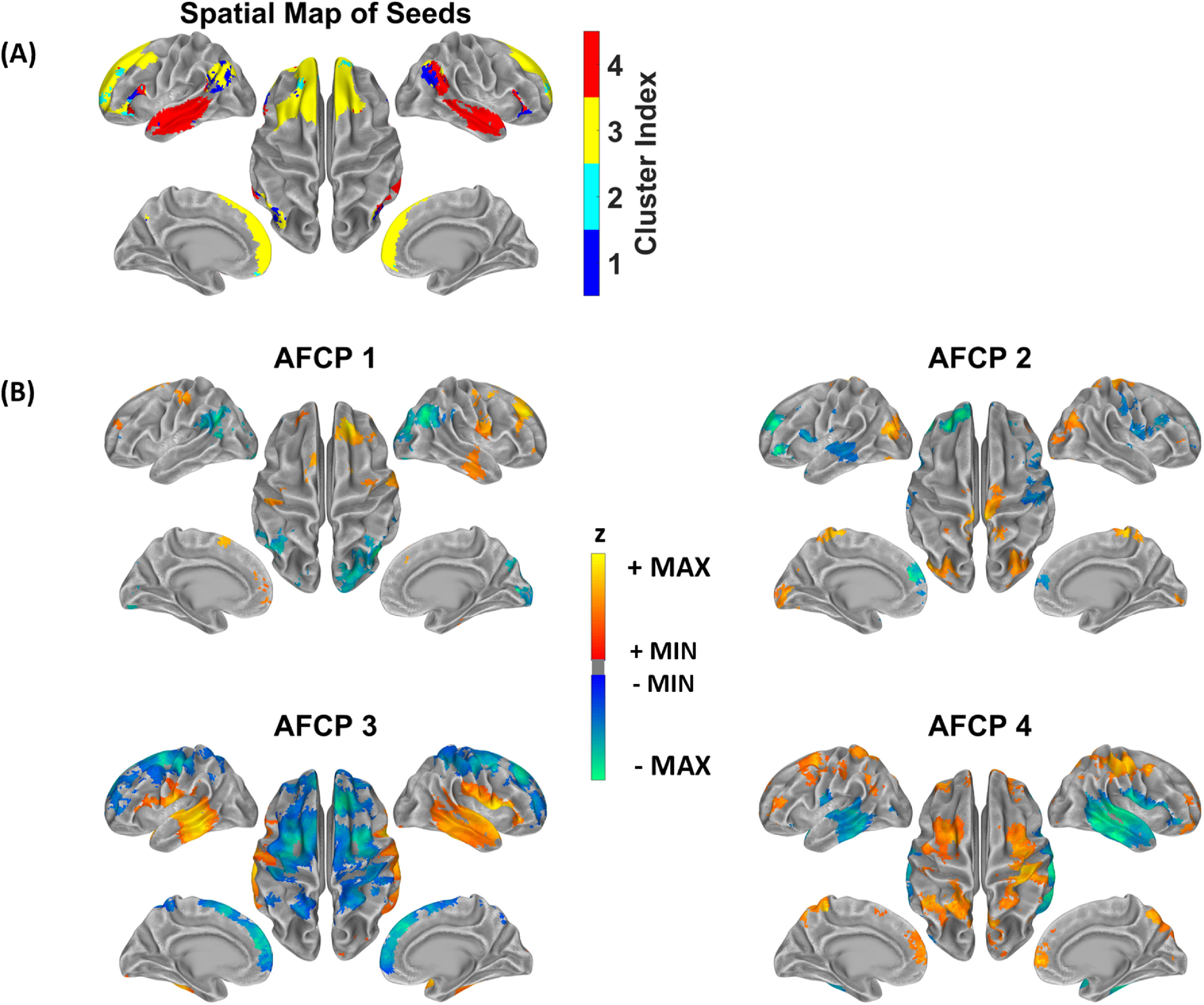
Aliased functional connectivity patterns (AFCP) after down-sampling. (A) Four AFCPs are discovered and their associated seeds. At each seed, the AFCP which has the highest number of sessions in all differential functional connectivity maps was assigned. (B) Spatial distribution of each AFCP. All maps are thresholded according to *q* < 0.05 after multiple-comparison correction. Red–yellow colorbar indicates increase of functional connectivity after thresholding. Blue–green indicates decrease of functional connectivity caused by the insufficient sampling frequency, after thresholding. The thresholded connectivity maps are overlaid on the standard cortical surface with grey colors indicating the curvature profile of the surface.

In the next step, we examined whether aliased activity due to insufficiently low sampling rate led to aliased functional connectivity. By exploring a data-driven analysis on the differential connectivity maps between DOT and DS-DOT, we have identified a set of four structurally organized patterns that represent the aliased connectivity. Figure 4 shows the spatial maps of seeds and centroids of the *AFCP* generated by the two-level *k*-means clustering. The numbers of clusters (*k*1 = 5, *k*2 = 4) were determined based on the L-curves of the ratio between within-cluster distance to between-cluster distance of the first- and second-level clustering, respectively (shown in supplemental figure 3). Figure 4(A) visualizes the spatial distribution of the seeds that drive each AFCP, via a winner-take-all strategy to map the seeds associated with the highest occurrence of clusters. Interestingly, for each AFCP, seeds that led to a common aliasing pattern exhibit as cortical patches with spatial continuity and bilateral hemispheric symmetry. For example, seeds for AFCP1 are aggregated at IPL, AFCP2 at prefrontal cortex, AFCP3 at mPFC and AFCP4 at temporal cortex, while all AFCPs involve seeds from bilateral hemispheres (figure 4(A)). Furthermore, AFCPs are distinct spatial patterns that include both increases and decreases in functional connectivity that are produced after down-sampling the signals to a low rate (figure 4(B)). Although the seeds were confined to be within the Yeo template of DMN, the aliased connectivity values extend to the whole cortical space. AFCP1 shows decreased functional connectivity in bilateral IPL and increased functional connectivity in frontal and visual cortices after down-sampling, while the AFCP1 is associated with seeds from IPL only. AFCP2 shows decreased functional connectivity in bilateral mPFC and temporal cortex and increased functional connectivity in bilateral visual cortex, with AFCP2 associated with seeds at prefrontal cortex. AFCP3 shows decreased functional connectivity in bilateral mPFC and motor cortex and increased functional connectivity in bilateral temporal cortex. Meanwhile, AFCP4 shows an almost reversed pattern against AFCP3, with increased functional connectivity in bilateral mPFC, motor cortex, IPL and precuneus and decreased functional connectivity in bilateral temporal cortex. Interestingly, despite their inversely related patterns, AFCP3 and AFCP4 are associated with distant seed patches, centering at mPFC and temporal cortices respectively.

**Figure 5.**
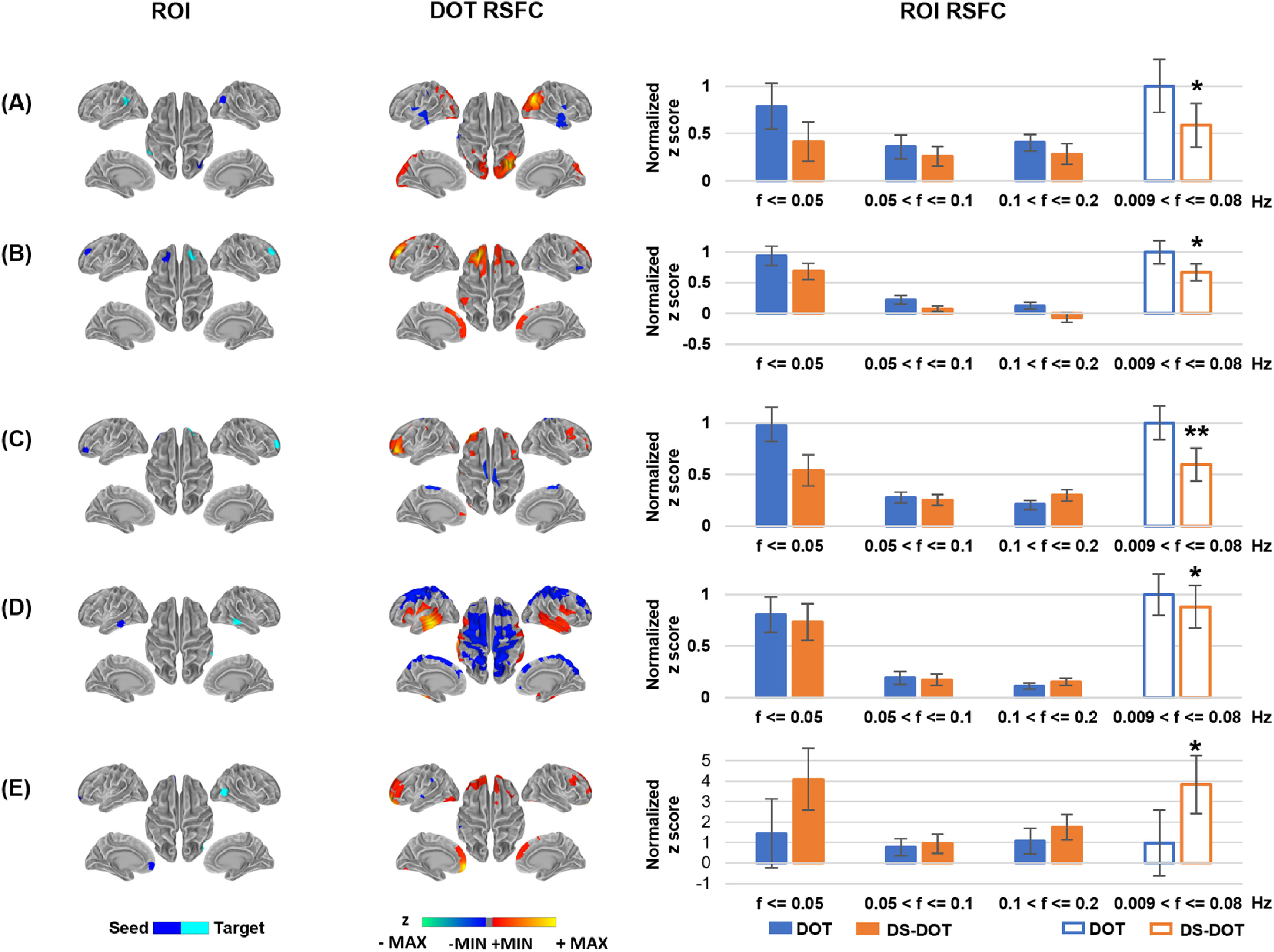
Frequency contributions to resting state functional connectivity in ROIs. Rows (A)–(D) present representative seed and target regions selected to show a decrease in functional connectivity in HbR DOT due to aliasing effects. Row E presents representative seed and target regions to show an increase in functional connectivity in HbO DOT. In the maps of DOT RSFC, all connectivity values are thresholded according to *q* < 0.05 after multiple-comparison correction. Walm colors from red to yellow indicate the positive *z* values after thresholding. Cold colors from blue to green indicate the negative *z* values after thresholding. The thresholded connectivity maps are overlaid on the standard cortical surface with grey colors indicating the curvature profile of the surface. In the bar graphs of ROI RSFC, the connectivity values (mean and standard errors) are normalized to the mean connectivity of DOT in the range from 0.009 Hz to 0.08 Hz, per each ROI pair. * indicates a significant difference in the DS-DOT than the DOT, at *p* < 0.05. ** indicates a significant difference in DS-DOT than DOT, at *p* < 0.01.

In order to further delineate the frequency-specific contributions of the aliased connectivity values, we have utilized a ROI approach to visualize the connectivity values before and after down-sampling. Figure 5 illustrates the seed and target ROIs circumscribed at hemispherical symmetric regions per each AFCP. Since RSFC are calculated in the filtered signals of 0.009–0.08 Hz as commonly in fMRI, the RSFC of original DOT of 0.009–0.08 Hz served as the normalizing baseline to compare the values across all frequency bins. Regarding the left and right IPL as representative ROIs (figure 5(A)), down-sampling has induced a significant decrease in the RSFC of 0.009–0.08 Hz. Meanwhile, the original DOT shows lower connectivity values in the upper frequencies (0.05–0.1 Hz and 0.1–0.2 Hz) that are below the bar at the ultra-slow frequencies (<0.05 Hz), suggesting that unsynchronized activities in upper frequencies are associated with the RSFC decreases after down-sampling. Figures 5(B)–(D) show additional ROIs at hemispheric symmetric positions in other AFCP, where the DS-DOT data present RSFC decreases of 0.009–0.08 Hz. Likewise, relative lower connectivity values are seen in the upper frequency range (0.05–0.1 Hz and 0.1–0.2 Hz), compared to those in the ultra-slow range (<0.05 Hz), again indicating the unsynchronized activities coincide with decreases of RSFC. In the contrary, increase of connectivity do occur in the down-sampled DOT as shown in another representative seed ROI at the mPFC (figure 5(E)). In this case, the upper frequencies 0.05–0.1 Hz and 0.1–0.2 Hz show DOT connectivity values as high as those in ultra-slow range (<0.05 Hz), which is noted for presenting much higher upper-frequency connectivity than other ROI of figures 5(A)–(D). Interestingly, accompanying such a strong connectivity in the higher frequency components is a multi-fold increase of connectivity in the DS-DOT. Notably, in the range of <0.05 Hz, the DS-DOT has reached almost *four folds* than DOT. These results suggest that the strong connectivity that already exists in the upper-frequency components may leak into in the ultra-slow range at an insufficient sampling rate, by bringing in aliased and substantially amplified contributions.

## 4. Discussion

In this study, we have demonstrated the feasibility of whole-head, cap-based and high-density fNIRS system in mapping large-scale DMN using conventional seed-based RSFC analysis. Previous fNIRS studies with partial or sparse montages have been able to map the RSFC concerning prefrontal (Mesquita *et al* 2010), sensorimotor (White *et al* 2009, Lu *et al* 2010, Mesquita *et al* 2010), auditory (Lu *et al* 2010) and visual areas (White *et al* 2009, Mesquita *et al* 2010). However, the capability of fNIRS in mapping large-scale brain networks has not been fully established, especially in those cognitively related brain networks such as DMN, dorsal attention network and frontoparietal control network that involve multiple distanced regions (Andrews-Hanna *et al* 2007, Greicius 2008, Zhang and Raichle 2010). Recent fNIRS studies have made progress in mapping part of DMN (Eggebrecht *et al* 2014), yet important midline regions or IPL are still missing due to lacking optodes to fully cover those. Here we reported a novel fNIRS–DOT study based on a whole-head and cap-based fNIRS array. Especially, our tomographic fNIRS data as illustrated in figure 3 have for the first time shown a complete DMN system including mPFC, posterior midline, and bilateral IPLs, which exhibits remarkable spatial similarity to fMRI RSFC maps derived from the same group of participants. In addition, the maps from fNIRS data also show correspondence with the template DMN generated in a large-scale fMRI study (Yeo *et al* 2011). The cross-model evaluation between DOT and fMRI has now included midline regions of DMN, which were missing in previous literature (Eggebrecht *et al* 2014). The seed-based approach employed in the current investigation allowed a direct comparison between fMRI and DOT in the same cohort of subjects, while the work by Khan *et al* (2021) used a data-driven approach and only reported the DOT results. Nonetheless, the network patterns derived from seeded nodes of DMN shown coherent organization with those from a data-driven approach.

Our demonstration of a fNIRS system in imaging DMN is especially important for monitoring the brain at aging and progressive AD states. Our results have revealed the functional connectivity among mPFC, precuneus and IPL, all of which are closely associated with memory and other cognitive functions (Sperling *et al* 2010). The inclusion of these key regions in the DMN has significant clinical values. Andrews-Hanna *et al* (2007) have shown that the anterior–posterior functional connectivity between midline DMN structures at semantic classification and passive fixation tasks is reduced in normal aging and positively correlated with cognitive performance. Similarly, Damoiseaux *et al* (2008) demonstrated that, in the resting state, the reduced RSFC in DMN is associated with aging years and correlated with declined performance in neuropsychological tests. A number of studies across individuals with AD or prodromal AD have documented altered functional connectivity within DMN, including precuneus/PCC, mPFC and inferior parietal cortex (Greicius *et al* 2004, Sorg *et al* 2007, Zhou *et al* 2010, Koch *et al* 2012). Intriguingly, it has been noted that the amyloid-*β* accumulation distribution in AD strikingly overlaps with the cortical hubs of high activity and metabolism (Buckner *et al* 2009), including mPFC, PCC and left/right IPL, which indicates the association between the functional connectivity at these cortical hubs and amyloid-*β* deposition (Buckner *et al* 2009). It is further postulated that the pathological process in these key DMN regions also extends to the aging individuals at the preclinical stage of AD even before clinical symptoms develop (Sperling *et al* 2009, Bateman *et al* 2012). Therefore, neuroimaging of such resting-state connectivity in DMN and other cognitive networks has been placed at pivotal positions, such as biomarkers, in clinical trials of intervention and prevention studies (Cavedo *et al* 2014, Sperling *et al* 2014). However, notably fMRI has been the primary modality, in many cases the only modality, to be included for functional neuroimaging in these studies. Power analysis for the prevention trials in AD usually yielded more than 1000 individuals are needed (Hsu and Marshall 2017). Considering the overwhelming number of people that are projected to be with AD and an even larger number to be placed at the prevention and intervention stages, the limited accessibility and high expenses of MRI instrument become a bottleneck in the workflow. Therefore, the fNIRS–DOT capability as demonstrated in our study provides an economic and practically feasible option for assessing the anterior–posterior DMN connectivity as biomarkers for monitoring aging and AD in large populations.

In addition, we have shown that a sampling rate that is much lower than the respiration and cardiac pulse frequency will introduce spatially organized aliased connectivity to RSFC. By deliberately down-sampling the raw fNIRS recordings to a sub-hertz frequency, we demonstrated that differences in the activity as well as connectivity do occur in the down-sampled data. Furthermore, our data-driven analysis on the differential RSFC maps between DOT and DS-DOT has revealed that the aliasing effect is seed-/region-dependent (e.g. AFCP3 vs. AFCP4), and that the resulted spurious connectivity exist within the DMN regions and extend beyond in other cortical areas, appearing as aliased networks (figure 4). Although fNIRS offers the advantage of high temporal resolution, maintaining a high sampling rate while using a large number of optodes can become a challenge, or sometimes compromise, for the deployment of full-head coverage and high-density measurement. Despite that a low sampling has been widely used in similar fMRI studies, our findings have emphasized that maintaining a sufficiently high sampling rate of fNIRS is crucial, especially when RSFC of DMN is considered as biomarkers. For example, the functional connectivity between IPL and mPFC has been shown to be associated with normal aging (Andrews-Hanna *et al* 2007) and AD (Koch *et al* 2012). However, we demonstrated that when the seed is placed at mPFC, the aliasing effects can cause inflated and deflated functional connectivity in temporal cortex and mPFC, respectively (AFCP3 in figure 4). Another example is AFCP1 in figure 4, where down-sampling has led to decreased connectivity in hemispherically symmetrical left and right IPL regions, which is consistent with the fMRI findings that the IPL connectivity is decreased in the down-sampled data than the original data at short repetition time (Golestani *et al* 2017). Our analysis of the frequency decomposition on these connectivity values (figures 5(A)–(D)) has pointed out that the unsynchronized upper frequency range is likely the origin of the alternations seen in down-sampled data in the 0.009–0.08 Hz. The aliased activity from the respirational and cardiac frequencies could become unsynchronized noises to attenuate the correlations in cerebral hemodynamics, which is also supported by Huotari *et al* (2019) when down-sampled fMRI data with TR = 3 s had significantly lowered correlation coefficients than raw data. Meanwhile, aliasing-related increases to RSFC is also possible, as shown in figure 5(E), which suggests that the physiological noises in synchronization may be a significant contributor or even dominating the RSFC in insufficiently sampled cerebral hemodynamics. Since the aliasing effects on RSFC may obscure our current understanding, accurate imaging without aliasing is especially important in understanding AD stages, because of the high comorbidity of vascular diseases in aging populations.

Furthermore, implications of the organized aliased patterns are relevant to other means of measuring the cerebral hemodynamics, such as fMRI. Typically, whole-brain fMRI has a TR of 2 s and can only theoretically sample the signals up to 0.25 Hz, which is unfortunately lower than the necessary Nyquist rate for respiratory (∼0.3 Hz) and cardiac (∼1 Hz) activities. Earlier fMRI studies have already shown that aliased activities do exist in the voxel-wise measurements (Cordes *et al* 2000, 2001). In addition, cardiac pulsation (Glover *et al* 2000, Chang *et al* 2009) and respiration (Birn *et al* 2006, 2008) are major origins for aliased activity in typical fMRI scans. However, the aliasing effects on functional connectivity are still poorly understood. Our study has taken a novel data-driven approach and revealed, for the first time, several whole-brain patterns attributed to the aliasing. The AFCPs discovered in our study are supported by a number of fMRI studies. Tong *et al* (2011) has demonstrated that the aliased cardiac signals appear in a spatial distribution that consists of dense arteries and large veins, which appears similar to AFCP3 and AFCP4. Other studies using ultrafast fMRI at TR = 0.35 s (Boubela *et al* 2013) or 0.1 s (Huotari *et al* 2019) also identified similar patterns associated with cardiac pulsation. Importantly, our analysis has pointed out that the aliasing could contribute to not only increases but also decreases of connectivity in a reversed spatial pattern, depending on the different seed regions (mPFC drives AFCP3 vs. middle temporal cortex drives AFCP4). Meanwhile, studies utilizing ultrafast fMRI has demonstrated that respiration affects prefrontal and occipital regions (Tong and Frederick 2014), which also appears in AFCP1 and AFCP2. Noteworthy, the standard normalization procedure for human brain imaging focuses on normalizing the brain tissues into a homogenous space. However, the anatomy of vasculature is outside of the scope of such normalization. Nonetheless, our data-driven analysis that focuses on segregating the differential connectivity maps was able to discover several organized aliased connectivity patterns at a group level. Interestingly, recent emerging evidence has demonstrated that BOLD spontaneous activity might exist in frequency bands above 0.1 Hz, the conventional cutoff for functional connectivity (Boubela *et al* 2013, Chen and Glover 2015, Jahanian *et al* 2019). Although it is not clear whether or to what extent these high-frequency connectivity patterns are driven by noises or neuronal activity, delineating aliasing contributions from valid neuronal-driven hemodynamics is crucial in understanding the functional organization of the human brain.

There are a few limitations in the current study that should be noted. Although in this study we demonstrated that DOT has the capability of measuring the cerebral hemodynamic signals in most of the superficial cortices, however, DOT in principle has a limited penetration depth (∼3 cm) (Strangman *et al* 2002, Dehghani *et al* 2009b) and is not suited to measure functional activity in the deep brain tissue. This can potentially hinder DOT from mapping RSFC across all hubs in RSN which comprise deeper brain tissues. In this study, we selected the seed within the precuneus of DMN, which allowed us to compare the DOT and fMRI RSFC maps. However, PCC, an important hub in DMN that is also connected to the precuneus, is likely not reachable by DOT due to its depth and has been excluded in the tomographic reconstruction. Similarly, other deeper structures in the medial prefrontal areas, such as the subgenual cingulate cortex that has been found to be disrupted in major depression (Greicius *et al* 2007, Fox *et al* 2012), is likely not reachable by fNIRS because of the limited penetration depth. Nevertheless, mPFC, precuneus and IPL are still very important hubs of DMN. Therefore, although restricted to the superficial cortex, fNIRS can still monitor the RSFC in a great portion of functional hubs of RSN and identify biomarkers for neurological disorders. Another limitation of the current study is the sample size of 13 subjects to be included in the group-level analysis. Although the number is empirically sufficient in reaching a group-level activation, it is only at the par bar with some of the early proof-of-concept studies (Brookes *et al* 2011, Eggebrecht *et al* 2014, Yuan *et al* 2016). Nonetheless, a larger sample sized study should be warranted to replicate the initial pilot study and further examine the robustness of these findings.

## 5. Conclusion

In summary, functional neuroimaging of resting-state brain networks especially the default mode network has been placed at pivotal positions—as biomarkers—in studies of aging and clinical trials for AD. However, notably fMRI has been the primary modality, in many cases the only modality, to be included for functional neuroimaging in these studies. Our study has demonstrated that the cap-based, brain-wide fNIRS recordings can map the RSFC in the posterior midline, prefrontal and parietal structures of the default mode network, in a consistent pattern with fMRI. Furthermore, the high sampling rate in fNIRS can prevent the physiological noises from contributing to the network organization in the cerebral hemodynamic measurements. fNIRS as a broadly accessible modality may be able to accelerate aging research, especially in large populations.

## Data Availability

The data generated and/or analysed during the current study are not publicly available for legal/ethical reasons but are available from the corresponding author on reasonable request.
